# First report of mitochondrial COI in foraminifera and implications for DNA barcoding

**DOI:** 10.1038/s41598-021-01589-5

**Published:** 2021-11-12

**Authors:** Jan-Niklas Macher, Jeremy G. Wideman, Elsa B. Girard, Anouk Langerak, Elza Duijm, Jamaluddin Jompa, Aleksey Sadekov, Rutger Vos, Richard Wissels, Willem Renema

**Affiliations:** 1 Naturalis Biodiversity Center, Marine Biodiversity, Leiden, The Netherlands; 2 Biodesign Center for Mechanisms of Evolution, School of Life Sciences, Arizona State University, Tempe, AZ USA; 3 Department of Ecosystem and Landscape Dynamics, Institute for Biodiversity and Ecosystem Dynamics (IBED), University of Amsterdam, Amsterdam, The Netherlands; 4 Hasanuddin University, Makassar, Indonesia; 5 ARC Centre of Excellence for Coral Reef Studies, Ocean Graduate School, The University of Western Australia, Crawley, Australia; 6 Institute of Biology, Leiden University, Leiden, The Netherlands

**Keywords:** Microbiology techniques, Sequencing, PCR-based techniques, Marine biology, Gene targeting

## Abstract

Foraminifera are a species-rich phylum of rhizarian protists that are highly abundant in many marine environments and play a major role in global carbon cycling. Species recognition in Foraminifera is mainly based on morphological characters and nuclear 18S ribosomal RNA barcoding. The 18S rRNA contains variable sequence regions that allow for the identification of most foraminiferal species. Still, some species show limited variability, while others contain high levels of intragenomic polymorphisms, thereby complicating species identification. The use of additional, easily obtainable molecular markers other than 18S rRNA will enable more detailed investigation of evolutionary history, population genetics and speciation in Foraminifera. Here we present the first mitochondrial cytochrome c oxidase subunit 1 (COI) gene sequences (“barcodes”) of Foraminifera. We applied shotgun sequencing to single foraminiferal specimens, assembled COI, and developed primers that allow amplification of COI in a wide range of foraminiferal species. We obtained COI sequences of 49 specimens from 17 species from the orders Rotaliida and Miliolida. Phylogenetic analysis showed that the COI tree is largely congruent with previously published 18S rRNA phylogenies. Furthermore, species delimitation with ASAP and ABGD algorithms showed that foraminiferal species can be identified based on COI barcodes.

## Introduction

Foraminifera are a species-rich phylum of rhizarian protists^1^. They are highly abundant in a wide range of primarily marine environments and play a major role in global carbon cycling^2,3^. Understanding the diversity and ecology of Foraminifera is critical for observing both past^4–6^ and recent^7,8^ changes in ecosystems. To date, about 9600 extant species of Foraminifera have been recognised^9^, and the orders Rotaliida and Miliolida are especially species-rich with > 3000 and > 1700 species, respectively. Species recognition in Foraminifera is based mainly on structural and morphological characters, but foraminifera display extensive ecophenotypic variation^10–12^, leading to an ongoing discussion on the value of morphological variation and the need for extensive molecular work^13–16^. In the past 25 years, genetic data have provided new insights into the higher classification of foraminifera^17–20^, genetic variability in populations^21,22^, and revealed cryptic diversity in widely distributed morphospecies^15,23,24^. However, only a single genetic marker, the nuclear 18S ribosomal RNA, is widely used and available for molecular analyses of Foraminifera. While the 18S rRNA contains variable sequence regions that allow identification of most foraminiferal species^13,25^, some species show minimal variability, hindering identification^26^. Other species have hypervariable 18S regions^15,23^ and show high levels of intragenomic polymorphisms, i.e., highly different variants of the 18S rRNA within single specimens, which is potentially due to the presence of multiple nuclei within the single foraminiferal cell or hybridization of closely related species^21,27^. These challenges have been tackled by the advent of molecular species delimitation approaches for Foraminifera based on molecular taxonomic units (MOTUs)^15^. Still, interpretation of foraminiferal 18S rRNA data remains challenging and can therefore impede the interpretation of the results on genetic variability or speciation.

The use of readily obtainable molecular markers other than 18S rRNA might allow for the investigation of evolutionary history, population genetics and cryptic speciation in Foraminifera^15,28^. However, despite the sequencing of genomes and transcriptomes^29–31^ and few phylogenetic studies using nuclear markers^32–34^, no genetic marker other than rRNA has been widely used for species identification in Foraminifera. Most animals, red algae and naked amoeba can be identified using the mitochondrial cytochrome c oxidase subunit I (COI) gene^35–37^. COI has also been shown to be helpful in identifying some microbial eukaryotes^38,39^, and might therefore be promising for studying Foraminifera. To date, however, no COI gene sequences of Foraminifera have been identified and published.

Here we present the first mitochondrial COI gene sequences of Foraminifera, and primers that allow amplification of a wide range of foraminiferal species. We obtained COI sequences of 49 specimens from 17 species from the orders Rotaliida and Miliolida and show that COI allows the identification of species. Furthermore, the availability of foraminiferal COI genes allows the deposition of Foraminifera in commonly used repositories for mitochondrial reference sequences like the Barcode of Life database (BOLD^40^), which are widely used for molecular community analyses^41,42^ and will help improve species identification in this genetically understudied, but diverse and globally important group of protists.

## Materials and methods

We analysed a total of 49 Foraminifera specimens from 17 morphospecies. Benthic Foraminifera were sampled from the Spermonde Archipelago in Indonesia and from Coral Bay in Australia. One species of planktonic foraminifera was collected from the North Atlantic Ocean, and one dataset was downloaded from the NCBI Sequence Read Archive (SRA). See Supplementary Table 1 for locations and sample details. All collected specimens were stored in > 90% ethanol after sampling and transferred to the Naturalis laboratory for morphological species identification and molecular analyses. Species were sorted into morphotypes, identified, and photographed using a Zeiss Discovery v12 stereo microscope.

### DNA extraction

Foraminifera specimens were dried in 1.5-ml Eppendorf tubes and broken into fine powder using a porcelain mortar and pestle. We performed total genomic DNA extraction using the QIAamp DNA Micro Kit (Qiagen; Hilden, Germany) following the manufacturer's protocol. After extraction, DNA quantification was conducted using the FragmentAnalyzer system (Agilent Technologies, Santa Clara, USA). Since the extracted DNA was already fragmented to less than 500 bp average length, we did not conduct further fragmentation using ultrasonication or enzymes.

### Single cell shotgun sequencing library preparation

We prepared single-cell shotgun sequencing libraries for 21 specimens from 8 species (see Supplementary Table 1) using the New England Biolabs NEBNext Ultra II DNA Library Prep Kit (Ipswitch, USA) with the corresponding NEBNext Multiplex Oligos for Illumina, following the manufacturer’s protocol but reducing volumes by 50 percent. Final concentration and fragment size were checked on the Tapestation system (Agilent Technologies, Santa Clara, USA). We pooled samples equimolarly before sending for sequencing on the Illumina NovaSeq 6000 platform (2 × 150 bp read length) at Baseclear (Leiden, The Netherlands).

### Bioinformatic analysis of shotgun data

We used MultiQC^43^ for quality assessment of raw shotgun reads, which were subsequently loaded into Geneious Prime (v.2020). Reads were mapped against COI sequences of the rhizarian *Lotharella oceanica* deposited in GenBank (accession number NC_029731.1^44^) with up to 49% mismatch, word length of 5, and up to 10% gaps (gap size 10) allowed. We chose this reference as no foraminiferal or closely related (e.g., Radiolaria) COI sequences are available. Regions with a high coverage (> 20) of mapped reads were manually inspected. Mapped reads from these regions were used as a reference for repeated mapping of shotgun reads with the Geneious mapper, with minimum 100 base pairs overlap, maximum 1% mismatch and no gaps allowed. Mapping was repeated until no further reads could be mapped. We mapped reads back against the obtained contigs to check coverage, and identified open reading frames (ORFs) with mitochondrial translation table 4, which has previously been reported for protist mitochondrial genomes^45^.

We submitted the contigs to the mfannot mitochondrial annotation server of the University of Montréal (https://megasun.bch.umontreal.ca/cgi-bin/mfannot/mfannotInterface.pl). ORFs identified as COI were searched against the NCBI GenBank reference database^46^ using blastn to identify ORFs stemming from putative symbionts or contamination and candidates for foraminiferal COI. Annotations were manually curated in Geneious. Putative foraminiferal COI ORFs, which we identified based on high coverage and a lack of closely related hits in reference databases, were translated to proteins, subject to transmembrane prediction with TMHMM^47^ and searched against Pfam^48^, UniProt^49^, SwissProt^50^ and Ensembl^51^ databases using the hmmer web server^52^.

To verify that foraminiferal COI can be obtained from a previously published dataset, we downloaded a *Globobulimina* (order Rotaliida) metagenome from the NCBI Sequence Read Archive (accession number: SRX3312059^53^) and followed the workflow described above. Furthermore, we downloaded the genomic contigs of *Reticulomyxa filosa*^29^ and *Astrammina rara*^30^ and searched for mitochondrial genes as described above.

### Amplification of foraminiferal COI

To test whether a COI “barcoding” fragment could be readily obtained without applying shotgun sequencing, we designed and tested eight primers based on the Leray-XT primers, which amplify a wide variety of eukaryotic taxa^54,55^. The original Leray-XT primers were mapped against the consensus of the shotgun sequenced and assembled Rotaliida and Miliolida COI sequences in Geneious Prime. We adjusted the primer sequences to fit all sequence variants found in the new target organisms. The newly designed primers are shown in Table 1. See Supplementary Material 2 for alignments of Foraminifera COI sequences and primers.

**Table 1:** Primer sequences designed for amplification of Foraminifera.

**Miliolida-specific**	
**Forward primers**	**Primer sequence (5′–3′)**
Miliolida_COI_fwd1	GGGAGGAGTTAATGCTGGTYG
Miliolida_COI_fwd2	AATGCTGGTYGAACWTTTTACGTACC
**Reverse primer**	
Miliolida_COI_rev	GAGCTTCAGGATGACTAAGAGATC
**Rotaliida-specific**	
Forward primer	
Rotaliida_COI_fwd	CTGGTTGAACATCTCATGCTC
Reverse primer	
Rotaliida_COI_rev	CTTCTGGATGTCTAAGAAATCAARG
**Rotaliida and Miliolida**	
**Forward primers**	
Foraminifera_COI_fwd1	GWGGWGTTAATGCTGGTYGAAC
Foraminifera_COI_fwd2	AATGCTGGTYGAACATYTYAYGYWCC
**Reverse primer**	
Foraminifera_COI_rev	RWRCTTCWGGATGWCTAAGARATC

We amplified COI for 36 specimens from 14 species of Rotaliida and Miliolida from the Naturalis collection (see Supplementary Table 1). Amplification of COI fragments was conducted with the PCR protocol shown in Tables 2 and 3. We amplified all specimens with the primer combinations “Foraminifera_COI_fwd1/Foraminifera_COI_rev” and “Foraminifera_COI_fwd2/Foraminifera_COI_rev”. Furthermore, we amplified Miliolida with the combinations “Miliolida_COI_fwd1/Miliolida_COI_rev” and “Miliolida_COI_fwd2/Miliolida_COI_rev”. Rotaliid specimens were further amplified with the primer combination “Rotaliida_COI_fwd/Rotaliida_COI_rev”. Amplified DNA was sent for Sanger sequencing at Baseclear (Leiden, The Netherlands). A negative control (sterile water) was processed together with the samples to check for potential contamination.

**Table 2:** Chemicals used for amplification of foraminiferal COI fragments; concentrations and volumes are shown per sample.

Chemicals	End concentration	Volume
MQ water		11.7 μl
PCR buffer CL	10×	2.0 μl
MgCl_2_	25 mM	0.4 μl
BSA	10 mg/ml	0.8 μl
Forward primer	10 pMol/μl	1 μl
Reverse primer	10 pMol/μl	1 μl
dNTPs	2.5 mM	0.4 μl
Qiagen Taq	5 U/μl	0.2 μl
DNA template		5 μl

**Table 3:** PCR protocol used for amplification of foraminiferal COI fragments.

Step	Temperature (°C)	Time	Cycles
Initial denaturation	96	3 min	
Denaturation	96	15 s	40
Annealing	50	30 s	
Extension	72	40 s	
Final extension	72	5 min	

### Bioinformatic analysis of barcoding data

We quality checked and assembled the obtained Sanger raw sequences in Geneious Prime, and MAFFT aligned and trimmed them to the same length (310 bp). In case the same specimen was successfully amplified with different primer pairs, we chose the sequence with the highest sequence quality for alignment and subsequent analyses. To assess whether COI barcodes allow identification of Foraminifera species, we calculated a tree with the IQ-Tree web server using default settings with 1000 iterations^56^. We assessed whether morphologically identified species form distinct clusters based on the amplified Leray COI fragment. *Calcarina hispida*, *Nummulites venosus* and *Globobulimina* sp. sequences of the same COI region, obtained only by shotgun sequencing and assembly, were added to this dataset to maximise species coverage. Furthermore, we applied the ASAP^57^ and ABGD^58^ (as commonly applied to Foraminifera^15^) species delimitation algorithms to the dataset, which can be used to identify putative species in datasets containing specimens of unknown identity or when little a priori information on species is available. We applied the Kimura-2-parameter (K2P) model, as it is proposed as the standard for DNA barcoding analyses^59^.

## Results

Single-cell shotgun sequencing and assembly resulted in complete mitochondrial COI sequences of 21 Foraminifera specimens from 8 species. In addition, we obtained a complete COI sequence from the *Globobulimina* dataset downloaded from NCBI SRA. Mean coverage ranged from 40.4 (sample “*Parasorites_sp*_3488”) to 895.6 (sample “*Amphisorus_sp*_3762”). See Supplementary Table 1 for coverage per sample. Transmembrane prediction and comparison with genes deposited in Pfam, UniProt, SwissProt and Ensembl databases confirmed that the closest match of the identified gene sequences with existing references is mitochondrial COI, and blast searches against NCBI Genbank revealed that the closest matches were COI sequences stemming from various eukaryotes. None of the available reference sequences showed pairwise identity above 78% with the newly generated foraminiferal COI sequences (see list of top matches in Supplementary Table 1). Open reading frame (ORF) annotation and subsequent MAFFT^60^ alignment of foraminiferal COI sequences revealed that all rotaliid species showed a single ORF corresponding to COI. All miliolid species showed a 2 bp deletion that resulted in a stop-codon in the COI protein translation. Manual insertion of N (representing a possible post-transcriptional modification) into the gap in the miliolid sequences resulted in one continuous ORF corresponding to COI, which could be translated into a complete COI protein sequence. No COI sequences were obtained from *Reticulomyxa filosa* and *Astrammina rara* datasets downloaded from NCBI SRA.

### Amplification of foraminiferal COI and species identification based on molecular “barcodes”

We designed new primers based on the Leray-XT primers^54^ (see Table 1) and amplified a fragment of the mitochondrial COI gene for 36 specimens from 14 species of Rotaliida and Miliolida from the Naturalis collection. Amplification failed for two species (*Calcarina hispida*, *Nummulites venosus*). The primer combination “Foraminifera_COI_fwd1/Foraminifera_COI_rev” resulted in the highest number of successful amplifications (11 species, 26 specimens). Furthermore, we amplified eight Rotaliida species (15 specimens) using the rotaliid specific primers "Rotaliida_COI_fwd/Rotaliida_COI_rev", and six miliolid species (17 specimens) using the miliolid specific primers “Miliolida_COI_fwd/Miliolida_COI_rev”. *Alveolinella quoyi* could only be amplified with the miliolid-specific primers. See Supplementary Table 1 for all amplification results.

**Figure 1:**
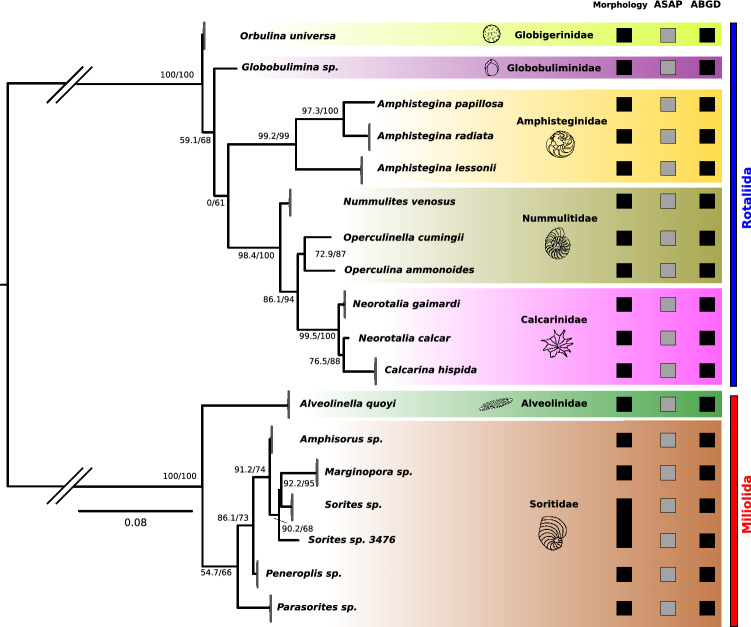
Phylogenetic tree showing evolutionary relationships of Foraminifera inferred from the Leray fragment of the mitochondrial COI gene. Numbers at nodes indicate bootstrap values (Maximum Likelihood) and posterior probabilities (Bayesian Inference). Branches within species are collapsed. Morphological identification and ASAP and ABGD delimitation results are shown. Squares indicate species/clusters identified based on morphology, ASAP and ABGD, respectively. Branches leading to Miliolida and Rotaliida, respectively, are shortened to improve readability.

All morphologically identified species formed distinct groups in the phylogenetic tree based on the Leray COI fragment, except for *Sorites* sp. (see Fig. 1). The tree revealed a similar topology as previously published 18S rRNA phylogenies of Foraminifera^18,20,61^. The orders Rotaliida and Miliolida form distinct clades. Within the Rotaliida, *Orbulina universa* (superfamily Globigerinoidea^9^, family Globigerinidae) is the sister clade of all other Rotaliida taxa in our study. *Globobulimina* (superfamily Serioidea^18^, family Globobulimidae) is the sister taxon of the clade comprising Amphisteginidae (superfamily Asterigerinoidea^9^), Nummulitidae (superfamily Nummulitoidea^9^) and Calcarinidae (superfamily Calcarinoidea^18^). The latter two form the sister groups of the Amphisteginidae, but with low support. In the Miliolida, *Alveolinella quoyi* (Alveolinidae, superfamily Alveolinoidea^9^) is resolved as the sister clade of the Soritidae (superfamily Soritoidea^9^). Within the Soritidae, *Parasorites* is the sister clade of *Peneroplis*, *Sorites*, *Amphisorus* and *Marginopora*. Within the latter clade, *Peneroplis* forms the sister clade of *Sorites*, *Amphisorus* and *Marginopora*. *Amphisorus* is the sister clade of *Marginopora* and *Sorites*. One Sorites specimen (“*Sorites* sp. 3476”), although morphologically identical with the other *Sorites* specimens, clusters as the outgroup of both *Marginopora* and the other *Sorites* specimens.

The ASAP species delineation algorithm^57^ reported two main clusters, corresponding to Rotaliida and Miliolida [asap-score 3.00, P-value 1.82e−01, threshold (genetic) distance 25%], with partitioning into 18 clusters/species receiving the second-highest score [ASAP score 3.50, P-value 2.87e−01, threshold (genetic) distance 0.7%]. These 18 clusters/species correspond to the morphospecies, except for specimen “*Sorites* sp. 3476”, which was delineated as a separate species/cluster. See Fig. 1 for ASAP results. Species delimitation with ABGD resulted in the same pattern found with ASAP, i.e., two main clusters corresponding to Miliolida and Rotaliida, respectively, and 18 clusters (with 0.77% reported interspecific genetic distance) corresponding to the identified morphospecies, except for the separately clustering “*Sorites* sp. 3476” (see Fig. 1).

## Discussion

We report the first published mitochondrial sequences and COI barcodes of Foraminifera, and primers that allow amplification of the Leray COI fragment for two major taxonomic orders, the Rotaliida and the Miliolida. Previous molecular work on Foraminifera has provided deeper insight into the group’s phylogeny and helped identify species. However, despite the sequencing of a limited number of genomes and transcriptomes^29–31^ and few phylogenetic studies using nuclear markers^32–34^, only one genetic marker, the nuclear 18S rRNA, is commonly available and used for molecular work on Foraminifera^18–20,23,25,28,61,62^. As Foraminifera can show high levels of intragenomic variability in this marker and highly variable rates of evolution are found in some genera and families, species identification and phylogenetic placement can be challenging^21,26,27,63^. Although approaches for molecularly identifying species and species groups based on 18S rRNA have been developed^15^, intragenomic polymorphisms in this marker can hamper inference of ecological and evolutionary patterns. Finding additional, easily obtainable genetic markers for Foraminifera is crucial for improving studies on the biodiversity and ecology of Foraminifera^13,64^, and including multiple markers into phylogenetic and ecological studies is becoming the standard in many fields of research^65–67^.

### Identification and amplification of foraminiferal COI

We morphologically identified 17 foraminiferal species from the order Miliolida and Rotaliida and used shotgun sequencing and assembly to obtain reference sequences for the commonly used mitochondrial “barcoding” gene COI. We subsequently designed primers and successfully amplified a fragment of foraminiferal COI for 14 species. For unknown reasons, amplification failed for two species of Rotaliida (*Calcarina hispida*, *Nummulites venosus*). Since the primer sequence matches the reference sequence obtained by shotgun sequencing of *Calcarina hispida*, we speculate that DNA quality or concentration was not sufficient for amplification. However, we cannot exclude that the amplification protocol and/or primers can be further optimised in future studies and for other Foraminifera species. Comparison with sequences in reference databases showed that the COI sequences we identified are not closely related to any previously published COI sequences. In combination with transmembrane prediction and comparison with genes deposited in Pfam, UniProt, SwissProt and Ensembl databases, as well as phylogenetic analyses, these findings led to the conclusion that we identified foraminiferal COI. We found that all analysed Miliolida species show a characteristic pattern of frameshifts or stop codon read-throughs in the COI gene, which will be of interest for future studies on mitochondrial evolution in Foraminifera. Previous studies on protist mitochondria have shown unique organisation, frameshifts, posttranslational modification and split genes in mitochondria of several protist taxonomic groups^68,69^, and this might also be the case in Foraminifera. As the same pattern was found in all analysed miliolids, we conclude that this is a unique feature of Miliolida mitochondria, which should be addressed in future studies.

### COI phylogeny of Foraminifera

We found a unique molecular COI “barcode” for all analysed species, thereby allowing species identification if suitable references exist. Our phylogenetic analyses of foraminiferal COI largely conform to previously published phylogenies based on 18S rRNA^18–20,61,62^. However, we point out that the phylogenetic tree shown in our study should be interpreted with care as it is based on the short (310 bp) Leray COI fragment and a limited number of taxa, with major groups like Textulariida and Monothalamea missing. The purpose of calculating the tree was to assess whether Foraminifera morphospecies fall into distinct clades based on the mitochondrial COI marker, i.e., whether species have unique COI “barcodes”.

Rotaliida and Miliolida form distinct, divergent clades, and within the Rotaliida, the Globigerinidae, Globobulimidae, Amphisteginidae as well as Nummulitidae plus Calcarinidae fall into separate clades that correspond to described superfamilies. The position of the Amphisteginidae within the Rotaliida is uncertain based on previously published 18S RNA data^61^, which showed weak support values, and the same holds true for our COI phylogeny. Future phylogenies should aim at including a higher number of marker genes to resolve the position of this taxon, which has been shown to be genetically highly diverse^23^.

Within the Miliolida, the Leray COI fragment resolves the Alveolinidae as the outgroup of the Soritidae, which is in line with 18S rRNA phylogenetic analyses^20^. Within the superfamily Soritoidea, previous phylogenies resolved *Peneroplis* (family Peneroplidae) as the outgroup of the Soritidae genera^20^, while our COI phylogeny resolved *Parasorites* (family Soritidae) as the outgroup of *Peneroplis* and the other Soritidae included in the analysis (*Amphisorus*, *Sorites*, *Marginopora*). However, Holzmann et al.^20^ found the position of *Parasorites* weakly supported and suggested that more research is needed. The specimen *Sorites* sp. 3476, which did not show morphological differences with the other analysed *Sorites* specimens, clustered as a sister taxon of both *Marginopora* and *Sorites* in the phylogenetic tree. Previous findings based on 18S rRNA sequences showed the genus *Sorites* to be paraphyletic and *Marginopora* branching within *Sorites*^20,34^. The genus comprises a high genetic diversity^70^, might contain a yet unknown number of cryptic species and should be revised using a combination of morphological and molecular work. Overall, we stress that our results on foraminiferal phylogeny based on the mitochondrial COI gene should be seen as preliminary, as the tree is based on a limited number of taxa, and we did not sequence species from the major groups Monothalamea and Textulariida. The latter cluster between the Rotaliida and Miliolida in 18S rRNA phylogenies^61^. Future studies should include more markers and more taxa to resolve higher levels of phylogeny and the phylogenetic position of families, general and species. Nevertheless, the availability of the commonly used mitochondrial barcoding gene COI can strengthen future phylogenetic analyses of Foraminifera by adding confidence through the number of studied genes.

### Automated species delimitation

We show that morphological identification and automated species delimitation based on molecular data are largely congruent. While 17 morphospecies were included in the dataset, automated species delineation with ASAP and ABGD resolved 18 clusters/species. The delineated clusters/species correspond to the morphospecies, except for one specimen of *Sorites* (sp. 3476), which also clustered as a sister taxon of both *Marginopora* and *Sorites* in the calculated phylogenetic tree (see “Discussion” above). Automated species delimitation can be used to identify Foraminifera species based on the Leray COI fragment, which might benefit applications like metabarcoding of community samples. These samples often contain many unknown taxa, for which reference sequences in databases are missing. Automated species delimitation can help estimate the number of species in such datasets and help define Molecular Operational Taxonomic Units (MOTUs). However, we point out that the species delimitation threshold identified by ASAP and ABGD was relatively low at about 0.7% interspecific genetic distance. Commonly used clustering approaches leading to the creation of molecular operational taxonomic units (MOTUs) based on fixed thresholds like 97% genetic identity could lead to an underestimation of species diversity when applied to Foraminifera COI datasets. Therefore, MOTU delineation approaches that combine intragenomic variability, intraspecific variability and prior taxonomic knowledge, which have been developed for 18S rRNA sequences of Foraminifera^15^, should also be explored for mitochondrial sequences by analysing larger datasets, including a high number of specimens per species. Furthermore, identification processes that take minor genetic differences into account, such as the Amplicon Sequence Variant (ASV) approach implemented in DADA2^71^ or the zero-radius OTU (ZOTU) approach implemented in UNOISE2^72^ should be considered in future (meta)barcoding studies on Foraminifera using the mitochondrial COI marker. Future studies including a large number of foraminiferal species will have to show whether closely related species can always be reliably distinguished based on the COI marker or if some species show either low variability or hypervariability, as reported for 18 s rRNA in some Foraminifera.

## Conclusion

Our study adds the first mitochondrial COI sequences of Foraminifera to databases and thereby makes mitochondrial barcoding available for further studies on this highly important group of marine protists. We show that shotgun metagenomic sequencing of genetically understudied taxonomic groups is a promising approach for identifying and developing novel mitochondrial markers. The build-up of reference databases containing foraminiferal COI and other genes combined with morphological identification of species is a crucial next step. Public sequence repositories containing 18 s rRNA of Foraminifera are available^13,16^, and adding more markers such as mitochondrial COI will allow adding Foraminifera to commonly used barcoding repositories like BOLD (Barcode of Life Database^40^) in addition to NCBI GenBank^73^. Future studies should broadly apply shotgun sequencing and molecular marker discovery to a wider set of rhizarian taxa in order to gain a better understanding of their mitochondrial diversity and evolution, and how these markers can be used to accelerate the identification of species in this highly important, yet understudied taxonomic groups.

## Supplementary Information

Supplementary Information.

Supplementary Table 1.

## Data Availability

Shotgun data is available from the NCBI SRA, BioProject PRJNA743004. All COI barcodes are available in NCBI GenBank, accession numbers: OL352650-OL352692, and Figshare: 10.6084/m9.figshare.16919071.v1.
