# Supplementary material for: First report of mitochondrial COI in foraminifera and implications for DNA barcoding
Source: Sci Rep. 2021 Nov 12;11:22165. doi: 10.1038/s41598-021-01589-5 (PMC8589990; doi:10.1038/s41598-021-01589-5)
Supplement: Supplementary file 1 — Supplementary Information. [file 41598_2021_1589_MOESM1_ESM.docx]

**First report of mitochondrial COI in foraminifera and implications for DNA barcoding**

Jan-Niklas Macher, Jeremy G. Wideman, Elsa B. Girard, Anouk Langerak, Elza Duijm, Jamaluddin Jompa, Aleksey Sadekov, Rutger Vos, Richard Wissels, Willem Renema

**Supplementary material 2**

# A)


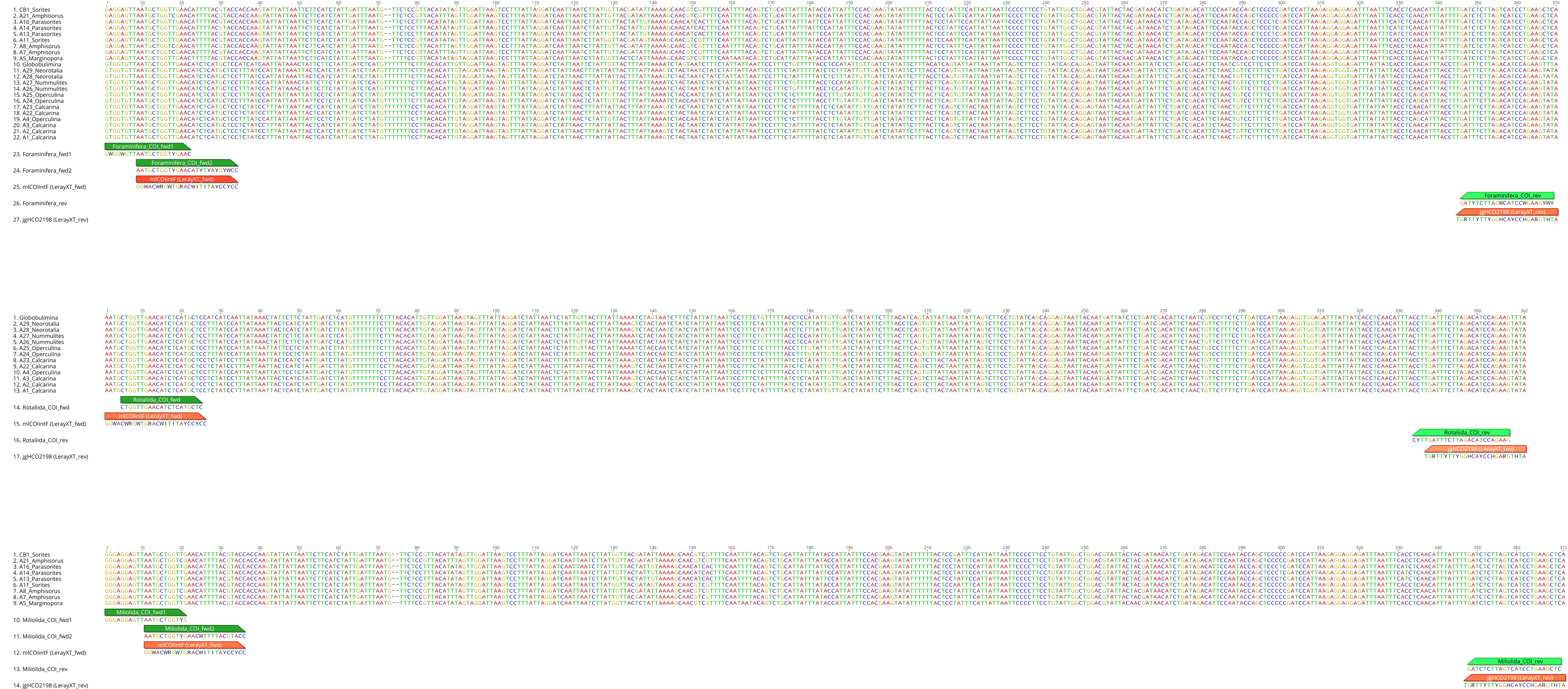


B)

# C)

**Supplementary material 2:** Alignments of foraminiferal COI sequences and newly designed primers for amplification of both Rotaliida and Miliolida (A), Rotaliida (B) and for Miliolida (C). Newly designed primers are highlighted in green. The LerayXT primers, which were used as reference for design of Foraminifera- specific primers, are highlighted in red.
